# Supplementary material for: Effects of Epoxyeicosatrienoic Acid-Enhancing Therapy on the Course of Congestive Heart Failure in Angiotensin II-Dependent Rat Hypertension: From mRNA Analysis towards Functional In Vivo Evaluation
Source: Biomedicines. 2021 Aug 20;9(8):1053. doi: 10.3390/biomedicines9081053 (PMC8393645; doi:10.3390/biomedicines9081053)
Supplement: Supplementary file 1 [file biomedicines-09-01053-s001.zip › Figure legends for supplementary figures.pdf]

Figure Legends

Supplementary Figure 1. Kidney mRNA expression of the renin-angiotensin system.

Kidney angiotensinogen (A), renin (B), angiotensin-converting enzyme (ACE) (C), ACE type 2 (ACE2) (D), angiotensin type 1 subtype A (AT_{1A}) receptor (E), angiotensin type 2 (AT_2) receptor (F) and Mas receptor (G) gene expression in sham-operated transgene-negative Hannover Sprague-Dawley (HanSD), sham-operated heterozygous Ren-2 transgenic rats (TGR) and TGR rats with aorto-caval fistula (ACF) two weeks after creation of ACF or sham-operation. * $P<0.05$ versus sham-operated HanSD rats.

Supplementary Figure 2. Kidney mRNA expression of the cytochrome-P450 enzymes and adrenergic receptors.

Kidney CYP2C23 (A), CYP4A1 (B), soluble epoxide hydrolase (sEH) (C), α1 subtype a (α1a) adrenergic receptor (D), α1 subtype b (α1b) adrenergic receptor (E) and β type 1 (β1) adrenergic receptor (F) in sham-operated transgene-negative Hannover Sprague-Dawley (HanSD), sham-operated heterozygous Ren-2 transgenic rats (TGR) and TGR rats with aorto-caval fistula (ACF) two weeks after creation of ACF or sham-operation.

Supplementary Figure 3. Biochemical parameters before initiation of treatment.

Plasma and kidney angiotensin II (ANG II) (A and E), plasma and kidney angiotensin-1-7 (ANG 1-7) levels(B and F), plasma and kidney ANG 1-7/ANG II ratios (C and G) and plasma and kidney norepinephrine levels (D and H) in sham-operated transgene-negative Hannover Sprague-Dawley (HanSD), sham-operated heterozygous Ren-2 transgenic rats (TGR) and TGR rats with aorto-caval fistula (ACF) two weeks after creation of ACF or sham-operation. * $P<0.05$ versus sham-operated HanSD rats.

Supplementary Figure 4. Assessment of protein expression (Western blot analyses) of the enzymes responsible for cytochrome P450 (CYP)-dependent production of eicosanoids before initiation of treatment.

Kidney and left ventricle CYP4A1 protein expression (A and E), kidney and left ventricle 20-hydroxyeicosatetraenoic acids (20-HETE) concentrations (B and F), kidney and left ventricle CYP2C23 protein expression (C and G) and kidney and left ventricle soluble epoxide hydrolase (sEH) protein expression (D and H) in sham-operated transgene-negative Hannover Sprague-Dawley (HanSD) rats, sham-operated heterozygous Ren-2 transgenic rats (TGR) and TGR rats with aorto-caval fistula (ACF) two weeks after creation of ACF or sham-operation. * $P<0.05$ versus sham-operated HanSD rats.

Supplementary Figure 5. Assessment of kidney tissue availability of metabolites of cytochrome P450 (CYP)-dependent epoxygenase pathway of arachidonic acid metabolism.

Kidney 5,6-epoxyeicosatrienoic acids (EETs) (A), kidney 8,9-EETs (B), kidney 11,12-EETs (C), kidney 14,15-EETs (D), kidney dihydroxyeicosatrienoic acids (DHETs) (E) in sham-operated transgene-negative Hannover Sprague-Dawley rats (HanSD), sham-operated heterozygous Ren-2 transgenic rats (TGR) and TGR rats with aorto-caval fistula (ACF) two weeks after creation of ACF or sham-operation. * $P<0.05$ versus sham-operated HanSD rats and sham-operated TGR.

Supplementary Figure 6. Assessment of left ventricle tissue availability of metabolites of cytochrome P450 (CYP)-dependent epoxygenase pathway of arachidonic acid metabolism.

Left Ventricle 5,6-epoxyeicosatrienoic acids (EETs) (A), left ventricle 8,9-EETs (B), left ventricle 11,12-EETs (C), left ventricle 14,15-EETs (D), left ventricle dihydroxyeicosatrienoic acids (DHETs) (E) in sham-operated transgene-negative Hannover Sprague-Dawley (HanSD), sham-operated heterozygous Ren-2 transgenic rats (TGR) and TGR rats with aorto-caval fistula (ACF) two weeks after creation of ACF or sham-operation. * $P<0.05$ versus sham-operated HanSD rats and sham-operated TGR.
