# Supplementary figures and images for: Effects of Epoxyeicosatrienoic Acid-Enhancing Therapy on the Course of Congestive Heart Failure in Angiotensin II-Dependent Rat Hypertension: From mRNA Analysis towards Functional In Vivo Evaluation
Source: Biomedicines. 2021 Aug 20;9(8):1053. doi: 10.3390/biomedicines9081053 (PMC8393645; doi:10.3390/biomedicines9081053)

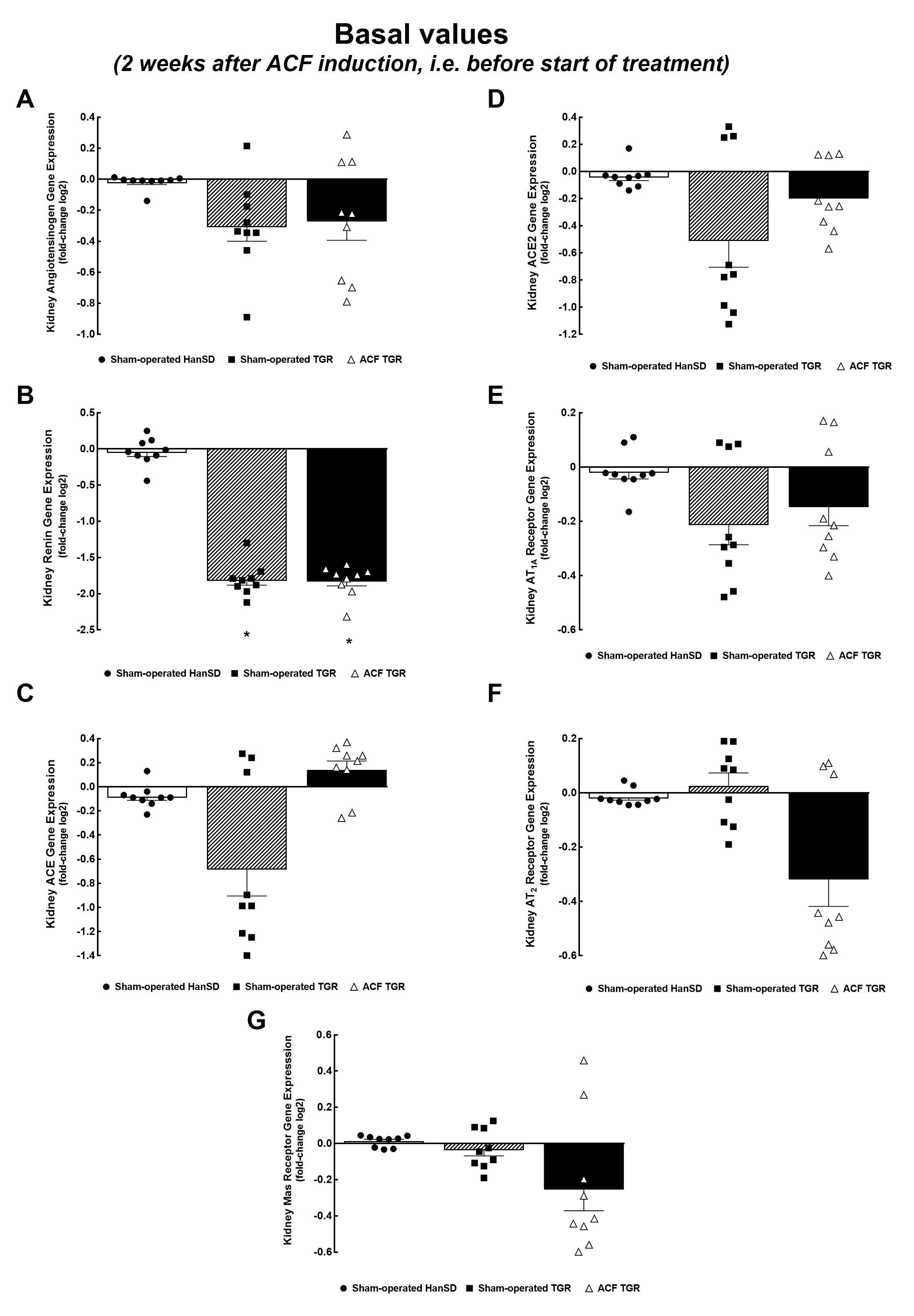

Supplement: Supplementary file 1 [file biomedicines-09-01053-s001.zip › Supplementary Figure 1.jpg]

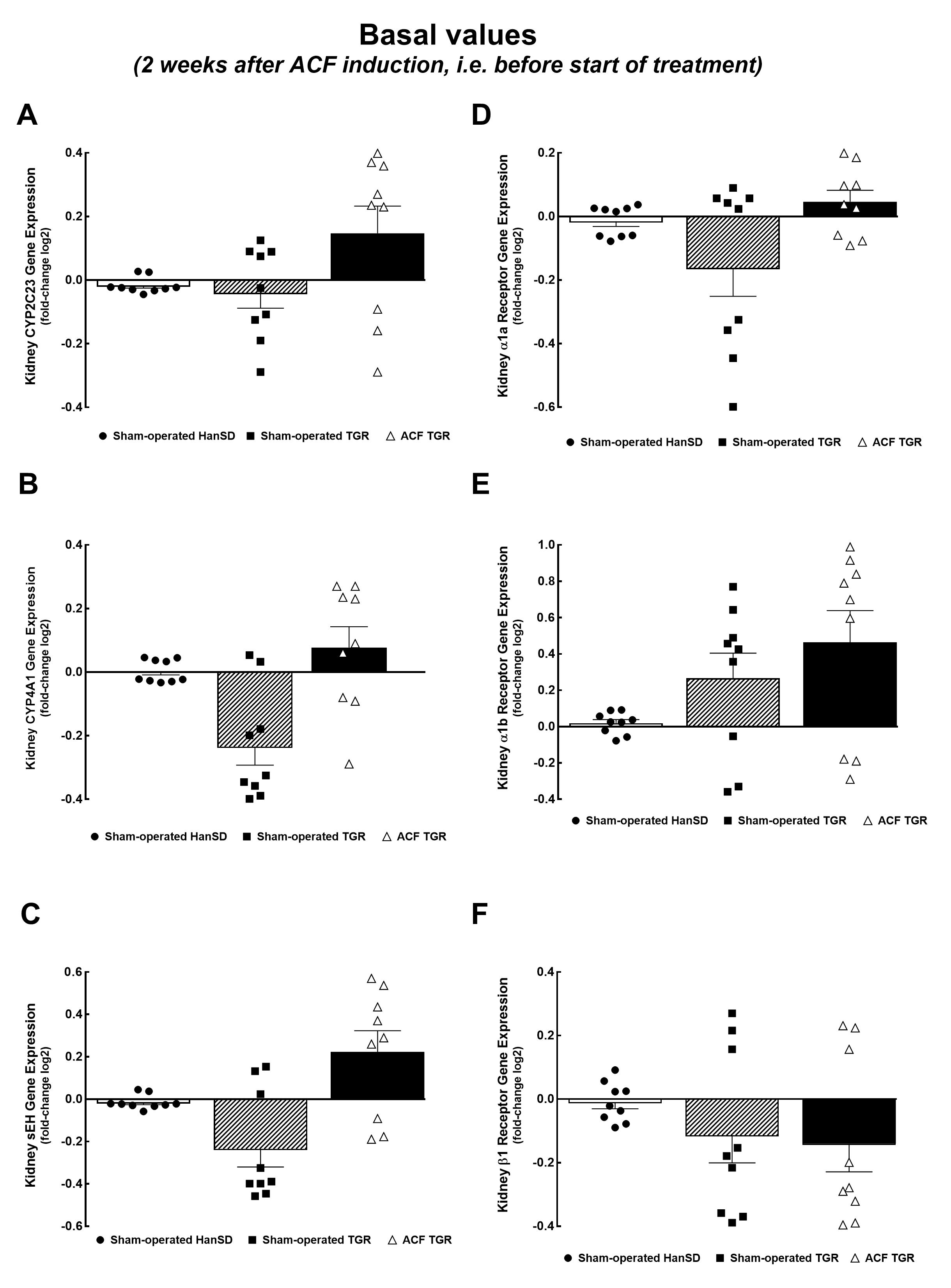

Supplement: Supplementary file 1 [file biomedicines-09-01053-s001.zip › Supplementary Figure 2.jpg]

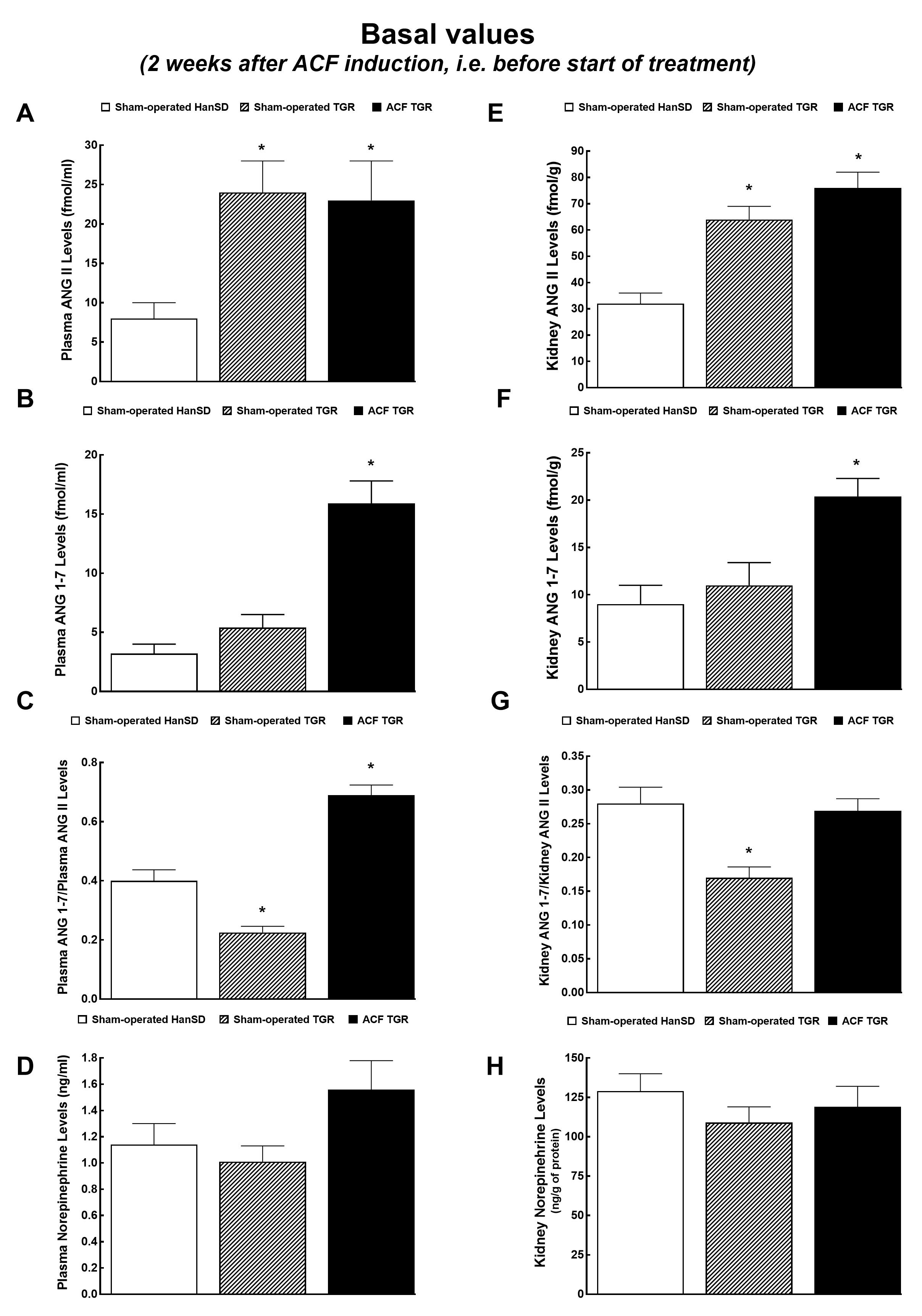

Supplement: Supplementary file 1 [file biomedicines-09-01053-s001.zip › Supplementary Figure 3.jpg]

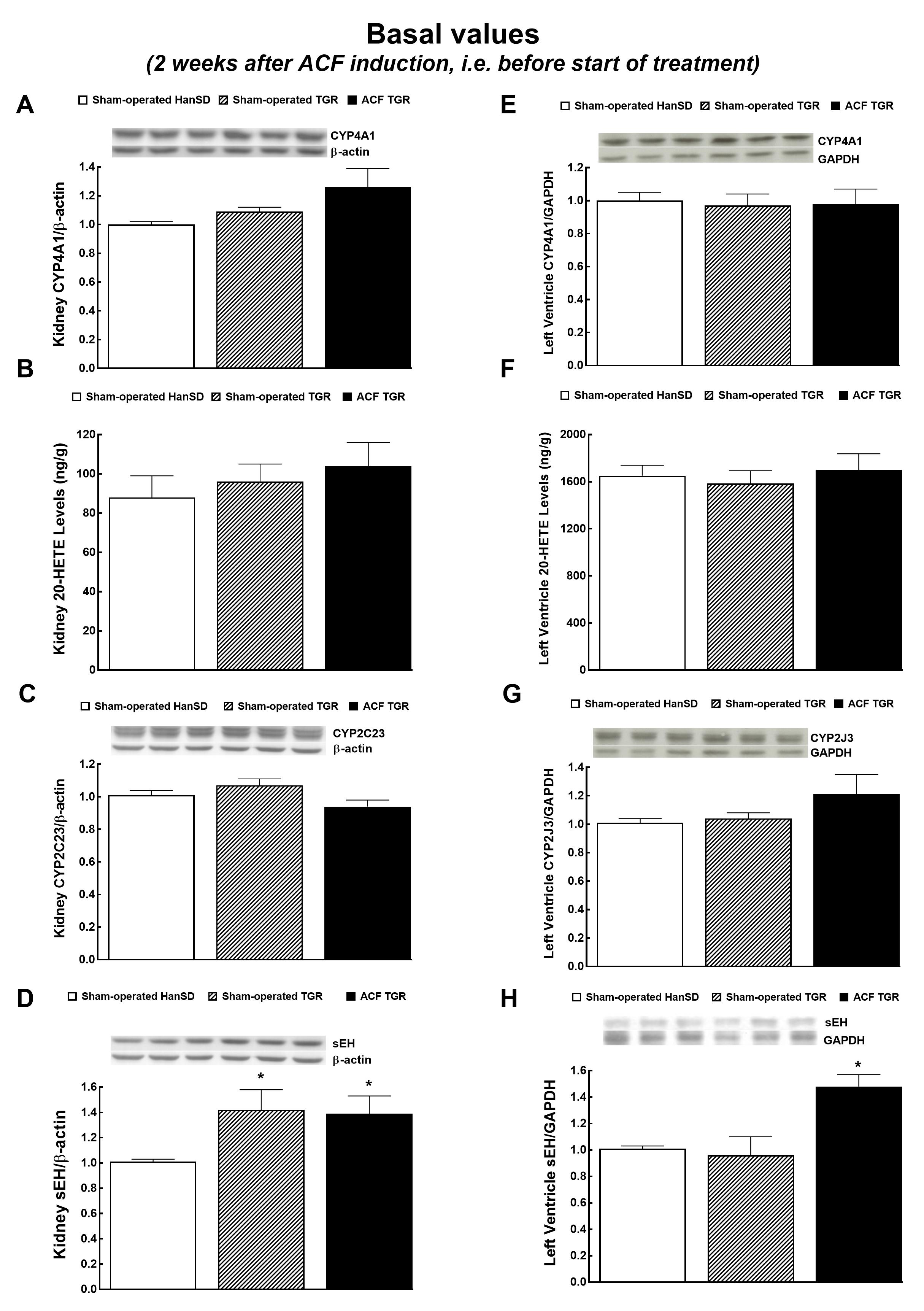

Supplement: Supplementary file 1 [file biomedicines-09-01053-s001.zip › Supplementary Figure 4.jpg]

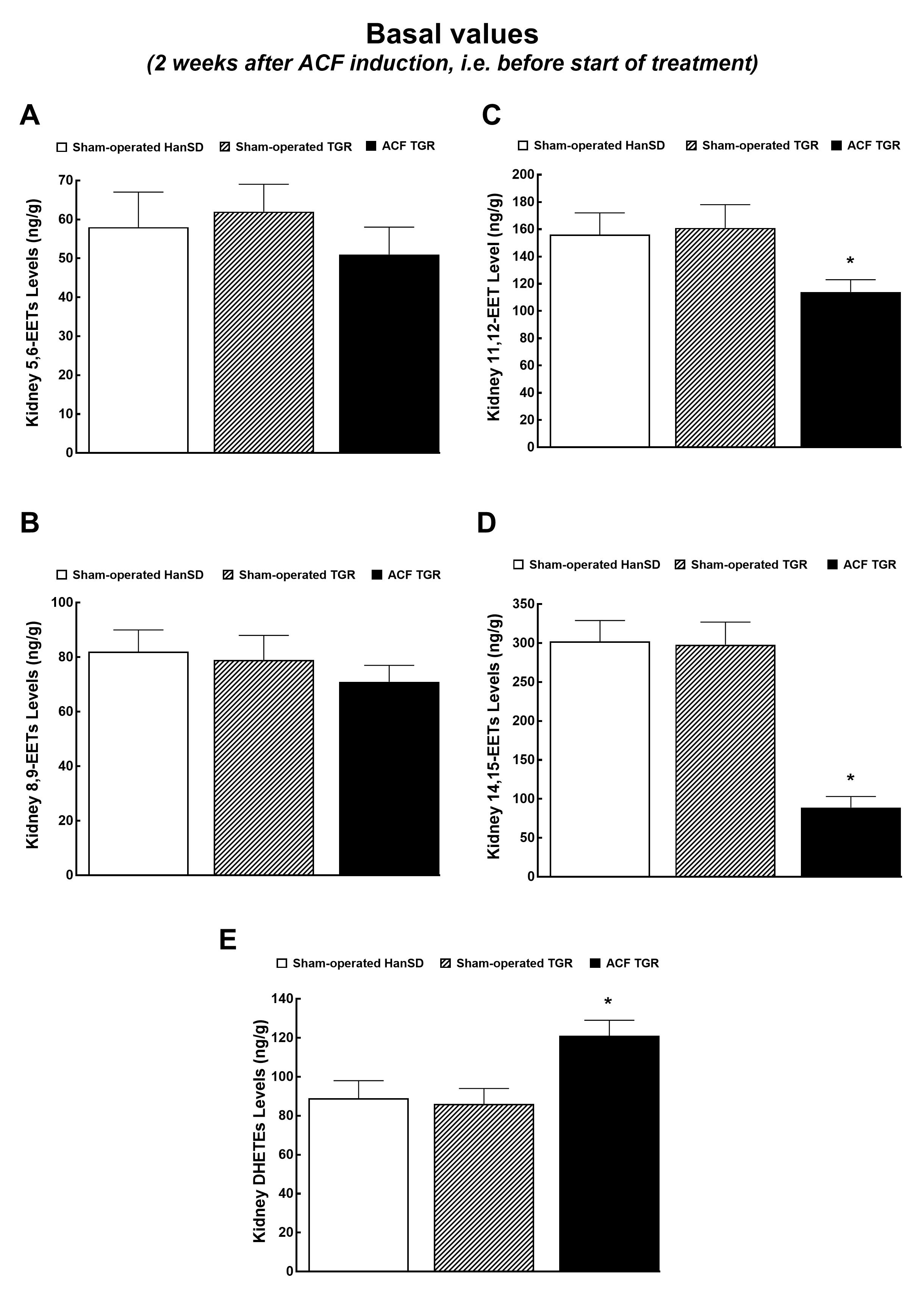

Supplement: Supplementary file 1 [file biomedicines-09-01053-s001.zip › Supplementary Figure 5.jpg]

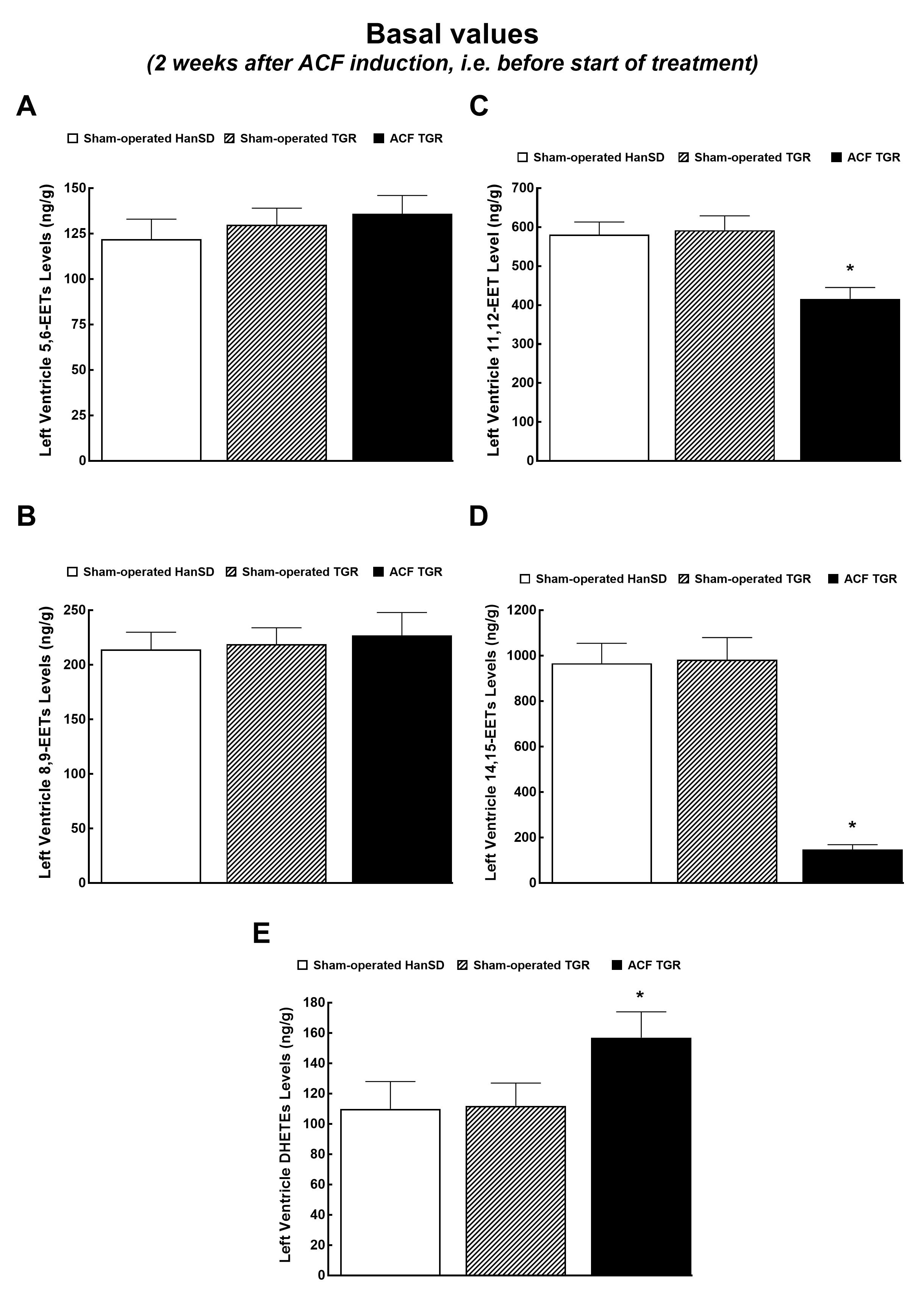

Supplement: Supplementary file 1 [file biomedicines-09-01053-s001.zip › Supplementary Figure 6.jpg]
